# Examining the impacts of 12 weeks of low to moderate-intensity aerobic exercise on depression status in patients with systolic congestive heart failure - A randomized controlled study

**DOI:** 10.6061/clinics/2019/e1017

**Published:** 2019-09-19

**Authors:** Walid Kamal Abdelbasset, Bader A Alqahtani, Ahmed A Elshehawy, Sayed A Tantawy, Tamer E Elnegamy, Dalia M Kamel

**Affiliations:** I Department of Physical Therapy and Health Rehabilitation, College of Applied Medical Sciences, Prince Sattam Bin Abdulaziz University, Alkharj, Saudi Arabia; II Department of Physical Therapy, Kasr Al-Aini Hospital, Cairo University, Giza, Egypt; III Department of Physical Therapy for Cardiovascular/Respiratory Disorder and Geriatrics, Faculty of Physical Therapy, Cairo University, Giza, Egypt; IV Department of Physical Therapy, Faculty of Applied Medical Sciences, Tabuk University, Tabuk, Saudi Arabia; V Department of Physiotherapy, College of Medical and Health Sciences, Ahlia University, Manama, Kingdom of Bahrain; VI Department of Physiotherapy, Centre of Radiation, Oncology and Nuclear Medicine, Cairo University, Giza, Egypt; VII Department of Physiotherapy for Women's Health, Faculty of Physical Therapy, Cairo University, Giza, Egypt

**Keywords:** Depression Disorder, Systolic Ccongestive Heart Failure, Aerobic Exercise, Patient Health Questionnaire-9

## Abstract

**OBJECTIVES:**

Psychiatric depression disorder is common in patients with systolic congestive heart failure (HF), and both conditions share underlying pathophysiological mechanisms. The incidence rate of depression disorder has clearly increased with the increase in HF manifestations in recent decades. Depression disorder is considered an independent predisposing factor for hospitalization, disturbed functional performance, and high rates of morbidity and mortality in HF patients. This randomized controlled study was designed to examine the impacts of low- to moderate-intensity aerobic exercise training on depression status in patients with systolic congestive HF.

**METHODS:**

A total of 46 systolic congestive HF patients with depression (40-60 years of age) were randomized to receive twelve weeks of mild- to moderate-intensity aerobic exercise plus standard medical treatment (exercise group) or standard medical treatment without any exercise intervention (control group). Depression status was examined using the validated Patient Health Questionnaire-9 (PHQ9) pre- and post-intervention at the end of the study program.

**RESULTS:**

No significant differences were observed between the exercise and control groups in demographic data or clinical characteristics (*p>*0.05). Both study groups showed a significant reduction in depression status at the end of the 12-week intervention (*p<*0.05). The comparison between the mean values of the depression scores showed significant differences between the two groups after 6 and 12 weeks of the intervention, indicating a greater reduction in depression scores in the exercise group than in the control group (*p*<0.05).

**CONCLUSIONS:**

Twelve weeks of a low- to moderate-intensity aerobic exercise program was safe and effective for reducing depression severity in patients with systolic congestive HF. Low- to moderate-intensity aerobic training should be recommended for cardiac patients, particularly those with HF-related depression.

## INTRODUCTION

The incidence of heart failure (HF) has increased in recent years in combination with the incidence of psychiatric depression disorder. HF is considered the main reason for critical morbidity and a major factor associated with hospital admission, increasing the mortality rate of the population 1. The main aim of HF management is to reduce symptoms, increase aerobic capacity, improve health-related quality of life (HRQoL), and overcome the restrictions induced by the disease 2,3. Some symptoms result from the low level of physical activity, which causes muscle fatigue and dyspnea; other symptoms are caused by a disturbed psychological status, such as that associated with depressive disorder, which leads to negative effects on HRQoL in patients with HF 4,5.

Recently, the prevalence of psychiatric depression has increased substantially in HF patients worldwide, ranging from 9-60% according to the characteristics of depression. The incidence rate of depression disorder ranges from 19.3-33.6% in patients with HF according to diagnostic questionnaires and personal interviews 6.

It was documented that depressive disorder is the factor that is most strongly associated with health risks, including functional dimension, increased symptoms of HF, and disturbance of HRQoL, in patients with HF. Furthermore, HF patients with depression have a higher risk for hospital readmission and death than non-depressed HF patients 7. Moreover, symptoms of HF are exacerbated by depression, particularly in HF patients with systolic hypertension 8.

To address the high incidence of depression among HF patients, exercise training may provide a safe solution, as antidepressant medications usually have undesired outcomes 9. Many studies have assessed the effect of aerobic exercise on cardiac disease 10-12, while few studies have explored the influences of exercise training on HF-related depression. Thus, the current study was designed to explore the impacts of low- to moderate-intensity aerobic exercise on depression status in patients with systolic congestive HF, hypothesizing that aerobic exercise training may have beneficial effects on depression status in HF patients.

## SUBJECTS AND METHODS

### Study design

A randomized controlled trial was designed to explore the effect of 12 weeks of low- to moderate-intensity aerobic exercise on the depression level of middle-aged patients with congestive HF. The committee for research ethics in the Department of Physical Therapy and Health Rehabilitation at Prince Sattam Bin Abdulaziz University approved the proposal [No.: RHPT/017/009]. All procedures of the study were executed in accordance with the ethical standards and guidelines of the Declaration of Helsinki 13. Each patient signed written informed consent before participating in the study program.

### Subjects

Forty-six eligible patients with systolic congestive HF (SCHF)-related depression were recruited for this study between June and September 2017. Their age ranged from 40 to 60 years, and they were diagnosed clinically with class II-III SCHF in accordance with the functional classes of the New York Heart Association (NYHA) regarding the severity of symptoms during physical activity 14.

The ejection fraction in all patients was <40% and was stable for one month without clinical complications; the patients were also diagnosed with depression (mild and moderate levels) according to reliably validated Patient Health Questionnaire-9 (PHQ-9) that incorporated depression diagnostic criteria 15.

All patients were referred by the medical psychologists of the university hospitals and were followed by cardiologists. Individuals were excluded from the study if they had a severe depression score, disturbed cognitive function, orthopedic complications, neuromuscular dysfunctions, or life-threatening diseases. Each participant was clinically cleared to participate in the exercise program.

### Power and sample size estimation

**Figure 1:**
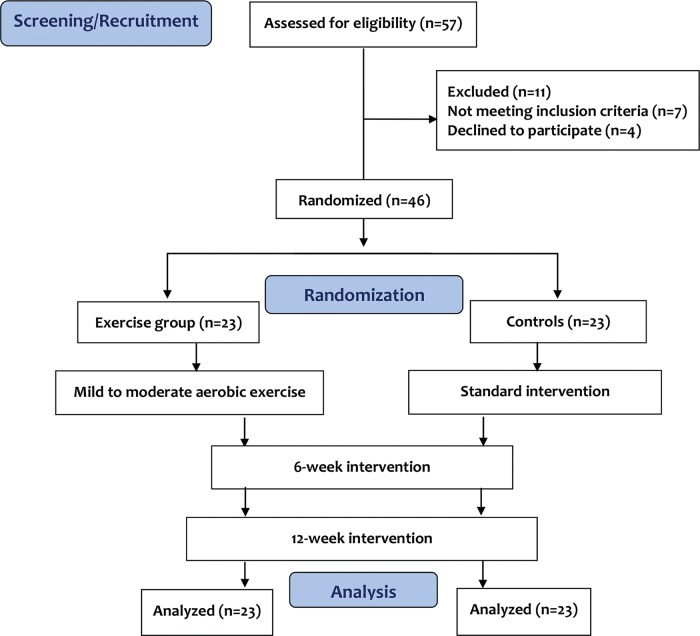
Flowchart of the study.

This randomized controlled study included forty-six patients due to the availability of resources rather than a power analysis. To avoid type II error, the 46 participants were randomized to exercise and control groups (23 in each group) using a random number table. The exercise group comprised twenty-three patients (16 men and 7 women) who performed low- to moderate-intensity aerobic exercise 3 times/week for 12 weeks. The control group comprised twenty-three patients (17 men and 6 women) who received only the standard medical treatment without any proposed exercise. Fifty-seven individuals were assessed for study eligibility. Eleven subjects were excluded (7 did not meet the inclusion criteria and 4 did not agree to participate in the study). The group allocation was determined before the initiation of the study program. The flowchart of the study is shown in Figure 1.

### Exercise protocol

The exercise group performed low- to moderate-intensity aerobic exercise training 3 times/week for 12 weeks. Each session began with five to ten minutes of warming-up and ended with five to ten minutes of cooling-down. A maximum heart rate (max HR) was estimated before starting the exercise program. During the first six weeks, the patients conducted low-intensity treadmill exercise at 40-50% of the max HR (20-30 min/session, 3 sessions/week). During the last six weeks, the patients conducted moderate-intensity treadmill exercise at 50-70% of the max HR (30-40 min/session, 3 sessions/week). All patients in the exercise group were supervised and received feedback to adjust the exercise intensity prescription. The control group was recommended to maintain their activity, to do fun tasks, to encourage the other subjects and to relax over the study period.

### Outcome measures

Depression status was assessed using the validated PHQ-9 at the beginning of the study program, after 6 weeks and after 12 weeks of the intervention at the end of the study by the same evaluator who was blinded to group allocation. The PHQ-9 was used to assess the severity of the depression status according to the score of the questionnaire. A score from 5-9 denotes mild depression, a score from 10-19 denotes moderate depression, and a score ≥20 denotes severe depression.

### Statistical analysis

The data were analyzed using the statistical package for social sciences (SPSS v.20, Chicago, IL, USA). The Kolmogorov-Smirnov test was used to assess the normality of the data. Categorical variables are reported as numbers and percentages, while the continuous variables are reported as the means ± standard deviations (SDs). Interferential statistics were used to evaluate the changes in depression scores utilizing unpaired *t*-tests between the exercise and control groups and one-way ANOVA between the three measures within each group. Statistical significance was set at *p*-value <0.05.

## RESULTS

Fifty-seven patients were screened for eligibility, eleven were excluded and forty-six individuals (33 men & 13 women) were included in the study program. Depression scores were normally distributed based on the Kolmogorov-Smirnov test. The results of the normality test of the PHQ-9 showed that the *p*-value=0.072, indicating a normal distribution of the data. No significant differences (*p*>0.05) were observed in any demographic data or clinical characteristics between the two groups before starting the study program (Table 1).

Depression status was significantly lower in the exercise and control groups (*p*<0.05) after 6 and 12 weeks of the intervention with significant differences between the two study groups (*p*<0.05). Before starting the study program, the mean depression score in the exercise group was 16.12±3.1, while in the control group, it was 15.95±3.14 (*p*>0.05). After 6 weeks of the intervention, the depression score decreased in the exercise group to 7.74±3.26 with a percentage change of 51.9%, while in the control group, it was reduced to 11.65±3.28 with a percentage change of 26.95%. After a 12-week intervention, the depression score decreased in the exercise group to 3.65±1.21 with a percentage change of 77.4%, while in the control group, it decreased to 8.54±2.14, with a percentage change of 46.46% (Table 2).

A comparison between the mean values of the depression scores showed significant differences between the two groups after 6 and 12 weeks of the intervention, indicating a greater reduction in depression status in the exercise group than in the control group (*p*<0.05), as demonstrated in Table 2.

## DISCUSSION

To the best of our knowledge, our present randomized controlled trial is the first study to examine the effects of low- to moderate-intensity aerobic exercise on depression status in middle-aged patients with SCHF, hypothesizing that adherence to aerobic exercise may reduce depression status in HF patients. Our data demonstrated that low- to moderate-intensity aerobic exercise was very effective in reducing depression scores in patients with SCHF.

Congestive HF is considered a common cause of psychiatric depression disease. To achieve ideal management of HF, the disturbed psychiatric condition must also be addressed 16.

Different studies have reported that physical exercise has beneficial effects on HF patients and controls the symptoms of psychiatric depression disorder and impaired mood. Further reports have proposed that aerobic exercise characteristics must be well structured, including exercise intensity, duration, and frequency, to achieve the desired reduction in the symptoms of major depressive disorder (MDD) 17.

It is widely accepted that aerobic exercise training increases the secretion of four molecules that reduce the impacts of MDD and lead to neurogenesis. Moreover, the positive clinical effects of exercise training on depressed subjects involve stress control, attitude improvement, outlook enhancement, self-dependence, confidence, trust and healthy mentality 15.

The present study showed that low- to moderate-intensity exercise training can provide a safe, realizable, and evidence-based physiotherapy practice with an advantageous culture for HF-related depression.

In our study results, it was documented that a 10-day walking exercise (thirty minutes/day) presented a definitive reduction in depression among individuals with MDD based on a depressive rating scale 18. Additionally, Knubben et al. showed that a short-term endurance exercise program resulted in a more substantial decrease in psychiatric depression disorder severity than antidepressant medications in patients with MDD 19. Moreover, prior studies have shown that exercise training under supervision had better effects on endurance capacity and energy consumption than home-based aerobics in patients with coronary artery disease and was also associated with a greater reduction in depression manifestations 20,21.

Our study has some limitations: 1) The present study did not verify the medical diagnosis of depressive disorder or HF disease in all patients in the study; and 2) Lack of some clinical and laboratory data, such as the etiology of HF disease and renal function. Future studies are also required to examine the impacts of different intensities and durations of aerobic exercise on HF-related depression.

## CONCLUSIONS

In summary, the 12-week low- to moderate-intensity aerobic exercise program was safe and effective for reducing depression severity in patients with SCHF. Furthermore, progressive aerobic training should be recommended for cardiac patients, particularly those with HF-related depression.

## AUTHOR CONTRIBUTIONS

Abdelbasset WK conceptualized and designed the study procedures, performed data curation and analysis, carried out the writing and editing of the original draft, and reviewed and confirmed the final submitted manuscript. Alqahtani BA contributed to the study procedure, data analysis, and the writing and editing of the original manuscript. Elshehawy AA contributed to the study procedures and data analysis, as well as, to the editing of the manuscript. Tantawy SA contributed to the data analysis, wrote the original draft, and revised and endorsed the final submitted manuscript. Elnegamy TE contributed to the data analysis, the writing of the original draft, and the review of the final submitted manuscript. Kamel DM contributed to the data curation, the writing of the original draft and the critical revision of the final submitted manuscript.

## Figures and Tables

**Table 1:** Demographic data and clinical characteristics of the two study groups.

Variables	Exercise (n=23)	Controls (n=23)
Age (years)	52.6±7.1	52.9±7.8
Sex, n (%)		
Males	16 (69.5)	17 (74)
Females	7 (30.5)	6 (26)
Education level, n (%)		
No formal education	3 (13)	3 (13)
Primary school	7 (30.5)	5 (21.7)
Middle school or more	13 (56.5)	15 (65.3)
Marital status, n (%)		
No	15 (65.2)	17 (74)
Yes	8 (34.8)	6 (26)
BMI (kg/m^2^)	29.8±2.8	30.2±2.4
Number of comorbidities	1.73±0.76	1.72±0.68
Clinical characteristics		
Ejection fraction (%)	34.2±4.8	36.3±3.4
VO_2_peak (mL min^−1^ kg^−1^)	15.42±3.4	14.85±3.7
Peak HR (bpm)	131±18	129±21
Peak SBP (mmHg)	177±26	173±22
Peak DBP (mmHg)	84±11	81±13

BMI, body mass index; VO_2_peak, maximal oxygen uptake; HR, heart rate; SBP, systolic blood pressure; DBP, diastolic blood pressure.

**Table 2:** Differences in the mean values of depression status before and after the treatment program (6 and 12 weeks) between the exercise and control groups.

Depression Scale	Exercise (n=23)	Controls (n=23)	*Sig.*
Before treatment	16.12±3.1	15.95±3.14	0.85
6 weeks posttreatment	7.74±3.26	11.65±3.28	<0.001
12 weeks posttreatment	3.65±1.21	8.54±2.14	<0.001
*Sig.*	<0.001	0.002	

Sig., significant level at *p*<0.05; Values are mean ± standard deviation.
